# Persistency of Cannabis Use Predicts Violence following Acute Psychiatric Discharge

**DOI:** 10.3389/fpsyt.2017.00176

**Published:** 2017-09-21

**Authors:** Jules R. Dugré, Laura Dellazizzo, Charles-Édouard Giguère, Stéphane Potvin, Alexandre Dumais

**Affiliations:** ^1^Centre de recherche de l’Institut Universitaire en Santé Mentale de Montréal, Montréal, QC, Canada; ^2^Department of Psychiatry, Faculty of Medicine, Université de Montréal, Montreal, QC, Canada; ^3^Institut Philippe-Pinel de Montréal, Montréal, QC, Canada

**Keywords:** persistency, cannabis use, violence, psychiatric patients, alcohol use, cocaine use, longitudinal study

## Abstract

**Background:**

Violence is a major concern and is prevalent across several mental disorders. The use of substances has been associated with an exacerbation of psychiatric symptoms as well as with violence. Compared to other substances such as alcohol and cocaine, existing literature on the cannabis–violence relationship has been more limited, with most studies being conducted in the general population, and has shown controversial results. Evidence has suggested a stronger relationship when examining the effects of the persistency of cannabis use on future violent behaviors. Though, while cannabis use is highly prevalent amid psychiatric patients, far less literature on the subject has been conducted in this population. Hence, the present prospective study aims to investigate the persistency of cannabis use in psychiatric patients.

**Method:**

The sample comprised of 1,136 recently discharged psychiatric patients provided by the MacArthur Risk Assessment Study. A multi-wave (five-assessment) follow-up design was employed to allow temporal sequencing between substance use and violent behaviors. Generalized estimating equations (GEE) were used to examine the effect of persistency of cannabis use on violence, while controlling for potential confounding factors. Potential bidirectional association was also investigated using the same statistical approach.

**Results:**

Our results suggest a unidirectional association between cannabis use and violence. GEE model revealed that the continuity of cannabis use across more than one time wave was associated with increased risks of future violent behavior. Patients who reported having used cannabis at each follow-up periods were 2.44 times more likely to display violent behaviors (OR = 2.44, 95% CI: 1.06–5.63, *p* < 0.05).

**Conclusion:**

These findings are particularly relevant as they suggest that the longer individuals report having used cannabis after a psychiatric discharge, the more likely they are of being violent in the following time waves. These results add to our understanding of the negative consequences of chronic cannabis use amid psychiatric patients.

## Introduction

Violence causes a serious public health burden (1). Literature has suggested that violence and criminality are greater in individuals with severe mental illnesses (SMI) when compared to the general population (2). Even higher rates of violence have been firmly established for those with substance use disorders (SUDs) (3). Moreover, substance use has been independently correlated with violence both in the general population and in the mentally ill (2, 4, 5). It is a great concern being associated with many adverse outcomes. It has been responsible for a great amount of the increased risk attributed to mental disorders (6–8) and has been linked to exacerbated psychiatric symptoms (e.g., psychotic symptoms) (9). While studies vary greatly, the general association between substance use, crime, and violence has long been established (3, 10, 11). Compared to other substances, the alcohol–violence relationship has been studied to a much greater extent both cross-sectionally and longitudinally (12–15). There is also quite strong evidence for the link between stimulants (i.e., cocaine and methamphetamine) and violence (3, 16).

However, while cannabis is a main substance of use in reports on arrests, violence, emergency room, and therapeutic admissions as well as involuntary injuries (17), the existing literature on its association with violence is more limited, notably amid psychiatric patients with most studies provided from the general population. Unlike studies of other drugs, the cannabis–violence relationship has provided controversial results (18). Cannabis use has often been found to have a positive link with aggressive behaviors both in cross-sectional and in longitudinal studies (18–24). For instance, results from the longitudinal Dunedin Study, which examined the relative risk for violence among different psychiatric disorders, indicated that after controlling for confounding variables such as other psychiatric disorders or other substance misuses (including alcohol dependence), marijuana-dependent individuals were 3.8 times more likely to report violence than controls (25). Cannabis use has been shown to be positively associated with violence among other studies on the mentally ill as well (26–28). While studies have shown a positive cannabis–violence association, a few have suggested more mixed results whereas its use was not related to violence or even suppressed it (18, 29). For instance, amid the psychiatric population, a lack of association was reported in a population of schizophrenia patients after the inclusion of other potential risk factors (30).

Several studies in the general population have also reported on the directionality of the cannabis–violence association as to whether involvement in violence may cause cannabis use or vice versa or whether they are intertwined in a more complex manner (31). Preliminary evidence nonetheless has suggested a dose–response relationship between cannabis smoking and violence/delinquency (32–36). Though, few longitudinal studies have been conducted, which are more suitable to examine causal relationships than cross-sectional studies (37). Moreover, some have more so examined a possible reciprocal relation between cannabis smoking and violence, further limiting the causal inferences that may be reached (35, 38–41). However, these analyses provided rather weak evidence. Interestingly, recent evidence has suggested a stronger relationship when examining the effects of the persistency of cannabis use on subsequent violent behaviors (32, 35, 41). Yet, only a limited amount of research has paid attention to this potentially important factor, none to our knowledge amid the psychiatric population.

Overall, no clear consensus has been established on the association and directionality of the cannabis–violence relationship. The main reason for these discrepant results may lie in the incongruent methodologies across studies (37) since they differ significantly with respect to measurement, developmental periods studied and the length of time between waves (42). Moreover, existing studies conducted have mostly been cross-sectional and may have been compromised by the likely presence of time-invariant (e.g., personality traits) and time-dynamic confounders (e.g., substance use) (43). In fact, the inclusion of covariates (confounders) known to influence both cannabis use and violence in statistical analyses, such as alcohol use or antisocial personality traits, has not always been reported (34). Studies that have not controlled for the effect of such variables may have overestimated the amplitude of the relationship between cannabis and violence. Indeed, a significant weakening of the association has been observed in studies that included these confounding variables. Furthermore, studies differ as to the population under study. Surprisingly far less attention has been directed toward investigating the relationship and directionality of the association as well as the association with persistent cannabis use amid psychiatric patients. Yet, cannabis use is known to occur more commonly in individuals with SMI (44).

Due to these gaps and the limited literature amid psychiatric population, this study aims to investigate the cannabis–violence relationship in a large sample of recently discharged psychiatric patients, with a focus on the persistency of cannabis use. To address several methodological limitations of prior literature, analyses of the data provided by the MacArthur Risk Assessment Study within a prospective design has been privileged. We hypothesized that a unidirectional association between cannabis and violence will be found and that persistency of cannabis use in individuals with mental health problems across time-points will be a significant predictor of subsequent physical assaults after controlling for important covariates of both cannabis use and violence revealed in the literature.

## Materials and Methods

### Study Sample

Participants were psychiatric patients recruited as part of the MacArthur Violence Risk Assessment Study (MVRAS). Between 1992 and 1995, data from 1,136 male and female patients were collected from 3 civil psychiatric facilities (Western Missouri Mental Health Center, Kansas City; Western Psychiatric Institute and Clinic, Pittsburgh; and Worcester State Hospital and the University of Massachusetts Medical Center, Worcester). After a complete description of the study, all participants provided their written informed consent. This study was approved by the local ethics committee from each site’s institutional review boards. A description of the MVRAS protocol has been detailed elsewhere (6, 45). Following hospitalization discharge, participants were interviewed every 10 weeks for a total of 5 different time waves. Of the 1,136 participants assessed at baseline, 846 were re-interviewed at time 1 (74.5%), 830 at time 2 (73.1%), 772 at time 3 (68.0%), 755 at time 4 (66.5%), and 754 at time 5 (66.4%).

### Study Design

To ensure temporal proximity, we examined the relationship between cannabis use [independent variable (IV)] and the presence of violent behaviors [dependent variable (DV)] in the following 10 weeks at each assessment. We used a prospective approach to test whether persistency of cannabis use across time-points would predict subsequent violent behaviors. First, cannabis use during the last follow-up period was coded dichotomously for each time-point (IV = non-user vs. user, 0-1) [e.g., (T1: 0-1), (T2: 0-1), (T3: 0-1), (T4: 0-1)]. Second, persistency of cannabis use was computed as a cumulative (ordinal) variable of cannabis use (dichotomous) across the following time waves [i.e., IV1 = T1 (0-1)], IV2 = T1 + T2 (0-1-2), IV3 = T1 + T2 + T3 (0-1-2-3), and IV4 = T1 + T2 + T3 + T4 (0-1-2-3-4). The same was performed for both cocaine and alcohol use.

For this study, violent behaviors were coded into a dichotomous variable (0/1) for each time-point to document the presence or absence of a violent behavior since the last follow-up. Table 1 describes the procedure used for our study’s design. Other potential confounding factors, such as psychopathic traits and impulsivity, were also considered in the statistical analyses.

**Table 1:** Description of the study’s design.

IV: Cannabis use (time-point)	DV: Violence outcome (time-point)
IV1: user vs. non-user = at (T1)	DV1: violence (T2)
IV2: user vs. non-user = at (T1) + (T2)	DV2: violence (T3)
IV3: user vs. non-user = at (T1) + (T2) + (T3)	DV3: violence (T4)
IV4: user vs. non-user = at (T1) + (T2) + (T3) + (T4)	DV4: violence (T5)

*IV, independent variable; DV, dependent variable; T, time point of assessment*.

### Assessments

#### Primary Diagnoses

Primary diagnoses were established after admission according to the DSM-III-R Checklist (46). Primary diagnoses included schizophrenia spectrum disorders, affective disorders, SUDs, and personality disorders.

#### Substance Use

At each time-point interview, participants were asked about their use of alcohol and other drugs since the previous follow-up period (in the prior 10 weeks). Since alcohol use and cocaine use are both known to be associated with cannabis use (47) and violence (3), we included these covariates in statistical analyses.

#### Psychopathic Traits

The Psychopathy Checklist Screening Version (PCL: SV) (48) contains 12 items assessing psychopathic traits. Each item is rated on a 3-point scale, with 0 being *non-applicable*, 1 being *possibly or partially present*, and 2 being *present*. The tool showed good psychometric properties among psychiatric patients (48, 49). As suggested by Hart et al. (48), we used a 3-point ordinal variable based on cut-off scores (total) in our analyses with psychopathic traits graded as the following: low psychopathic traits = total score ≤12; moderate psychopathic traits = total score <13–17; high psychopathic traits = total score ≥18.

#### Impulsivity

The Barratt Impulsiveness Scale [BIS-11; (50)] is a 30-item questionnaire developed to assess attentional impulsiveness (e.g., “I have racing thoughts”), motor impulsiveness (e.g., “I do things without thinking”), and cognitive/non-planning impulsiveness (e.g., “I say things without thinking”) in clinical and non-clinical populations. Each item is rated on a 4-point scale (1 = Rarely/Never, 2 = Occasionally, 3 = Often, 4 = Almost always/Always).

#### Outcome

At each time-point interview, the participants were asked questions about violent behaviors they committed in the past 10 weeks. Violence was assessed with the MacArthur Community Violence Instrument [MCVI; (45)], based on Lidz et al. (51), documenting aggressive behaviors, such as assaults, acts of battery, threats made with a weapon, used of a weapon against others, as well as rape.

### Statistical Methods

First, we examined the effect of persistency of cannabis use on violence using a generalized estimating equations (GEE) with a binomial distribution and a log link. Since substance consumption is time-dynamic; we, therefore, adjusted GEE on substance use with time. Multivariate analyses were then performed using the same approach adjusting for potential covariates such as time, age, sex, age at first psychiatric admission, other substance misuse, psychopathic traits, impulsivity, and primary diagnosis. The GEE is known to be flexible in handling correlated data structures (52), several outcome data types (e.g., binary, continuous), time-varying and time-invariant factors and missing data (53). The present sample size was large enough to estimate parameters with accuracy and, thus, to keep the stability of the working correlation matrix (54). Since cannabis use and violence were both coded dichotomously, we tested bidirectionality by using the same GEE model (with the same covariates) with a binomial distribution and a log link. Reverse directionality was tested by reversing variables (IV and DV) in multivariate models (i.e., cumulative violent behaviors on subsequent cannabis use) (35). Variables included in the models showed low levels of multi-collinearity [variance inflation factor (VIF) <2.0]. All statistical analyses were performed using SPSS for MAC version 23.

## Results

### Sample Characteristics

At baseline, the mean age was 29.74 years (SD = 6.24, range 18–40). The total sample included 1,136 participants, with a majority being men (58.7%), single, separated, or divorced (86.7%), Caucasians (69.3%) and mainly diagnosed with a primary affective disorder (52.6%). Schizophrenia spectrum disorders was diagnosed in 245 individuals (21.6%) of the sample, while 272 (23.9%) were primarily diagnosed with a SUD and 21 (1.8%) with a personality disorder. Moreover, the mean age at first psychiatric admission was 21.81 years (SD = 7.06) and most participants had a history of 4 or less psychiatric hospitalizations (66.0%). Participants reported having ever consumed alcohol (96.5%), cannabis (83.2%), or cocaine (59.2%) in their life.

The highest proportion of the total sample, that is 588 (61.9%) participants, was found to have never used cannabis at any of the time-periods. On the other hand, 157 (16.5%) participants consumed cannabis at 1 time-point, 77 (8.1%) at 2 time-points, 77 (8.1%) at 3 time-points, and 51 (5.4%) at 4 time-points. Violent behaviors were reported in 265 patients (23.3%) at time 1, 251 (22.1%) at time 2, 167 (14.7%) at time 3, 160 (14.1%) at time 4, and 136 (12%) at time 5.

### Persistency of Cannabis use and Risk of Subsequent Violence

As shown in Table 2, unadjusted GEE results revealed that the persistent use of cannabis significantly predicted violence occurring in the following time-point when comparing to no use of cannabis (one time-point: OR = 1.60, 95% CI: 1.19–2.15, *p* < 0.01; two time-points: OR = 1.91, 95% CI: 1.27–2.86, *p* < 0.01; three time-points: OR = 2.72, 95% CI: 1.66–4.46, *p* < 0.001; four time-points: OR = 4.04, 95% CI: 1.99–8.19, *p* < 0.001).

**Table 2:** Unadjusted generalized estimating equations with binomial distribution (log link) and an unstructured working correlation on violence occurring in the following time-points (*n* = 592).

	OR^a^ (95% CI)	*p*-Value
**Cannabis^b^**		
Use at 1 time-point	1.60 (1.19–2.15)	0.002
Use at 2 time-points	1.91 (1.27–2.86)	0.002
Use at 3 time-points	2.72 (1.66–4.46)	<0.001
Use at 4 time-points	4.04 (1.99–8.19)	<0.001
**Cocaine^b^**		
Use at 1 time-point	1.56 (1.12–2.17)	0.009
Use at 2 time-points	1.19 (0.75–1.90)	0.469
Use at 3 time-points	2.27 (1.49–4.80)	0.001
Use at 4 time-points	1.89 (0.75–4.76)	0.177
**Alcohol^b^**		
Use at 1 time-point	1.42 (1.07–1.89)	0.015
Use at 2 time-points	1.44 (1.02–2.04)	0.039
Use at 3 time-points	1.99 (1.30–3.04)	0.002
Use at 4 time-points	3.39 (1.99–5.78)	<0.001

*^a^Controlling for the effects of time*.

*^b^Ordinal variable (reference is no use of the substance across time-points)*.

Continuous use of alcohol across time-points statistically predicted future violent behaviors (one time-point: OR = 1.42, 95% CI: 1.07–1.89, *p* < 0.05; two time-points: OR = 1.44, 95% CI: 1.02–2.04, *p* < 0.01; three time-points: OR = 1.99, 95% CI: 1.30–3.04, *p* < 0.01; four time-points: OR = 3.39, 95% CI: 1.99–5.78, *p* < 0.001). However, only cocaine consumption at one and three follow-up periods were significant predictors of violence (one time-point: OR = 1.56, 95% CI: 1.12–2.17, *p* < 0.01; three time-points: OR = 2.27, 95% CI: 1.49–4.80, *p* < 0.01). For univariate analyses on potential covariates, see Table S1 in Supplementary Material.

The results from the GEE model suggest that the relationship between continued use of cannabis and subsequent violence remains relatively stable after adjustments for time-varying variables such as other substances use as well as fixed demographic and clinical covariates. In fact, as shown in Table 3, cannabis use over time was a significant predictor of violence when consumed at two time-points (OR = 1.71, 95% CI: 1.08–2.70, *p* < 0.05), three time-points (OR = 2.08, 95% CI: 1.16–3.74, *p* < 0.05), and four time-points (OR = 2.44, 95% CI: 1.06–5.63, *p* < 0.05).

**Table 3:** Generalized estimating equations with binomial distribution (log link) and an unstructured working correlation on subsequent violence (*n* = 592) adjusted for possible confounders.

	OR^a^ (95% CI)	*p*-Value
**Cannabis^b^**		
Use at 1 time-point	1.32 (0.94–1.86)	0.112
Use at 2 time-points	1.71 (1.08–2.70)	0.023
Use at 3 time-points	2.08 (1.16–3.74)	0.025
Use at 4 time-points	2.44 (1.06–5.63)	0.036
**Cocaine^b^**		
Use at 1 time-point	1.04 (0.71–1.53)	0.842
Use at 2 time-points	0.68 (0.41–1.14)	0.154
Use at 3 time-points	1.16 (0.59–2.28)	0.674
Use at 4 time-points	0.59 (0.21–1.63)	0.304
**Alcohol^b^**		
Use at 1 time-point	1.13 (0.84–1.52)	0.420
Use at 2 time-points	1.09 (0.74–1.61)	0.656
Use at 3 time-points	1.36 (0.84–2.21)	0.209
Use at 4 time-points	2.32 (1.25–4.28)	0.007

*^a^Adjusting for the effects of time, other substances used at each time-point, age, age at first hospitalization, sex, ethnicity, schizophrenia spectrum disorders (presence/absence), affective disorders (presence/absence), psychopathic traits (PCL), impulsivity (BIS-11)*.

*^b^Ordinal variable (reference is no use of the substance across time-points)*.

**Figure 1:**
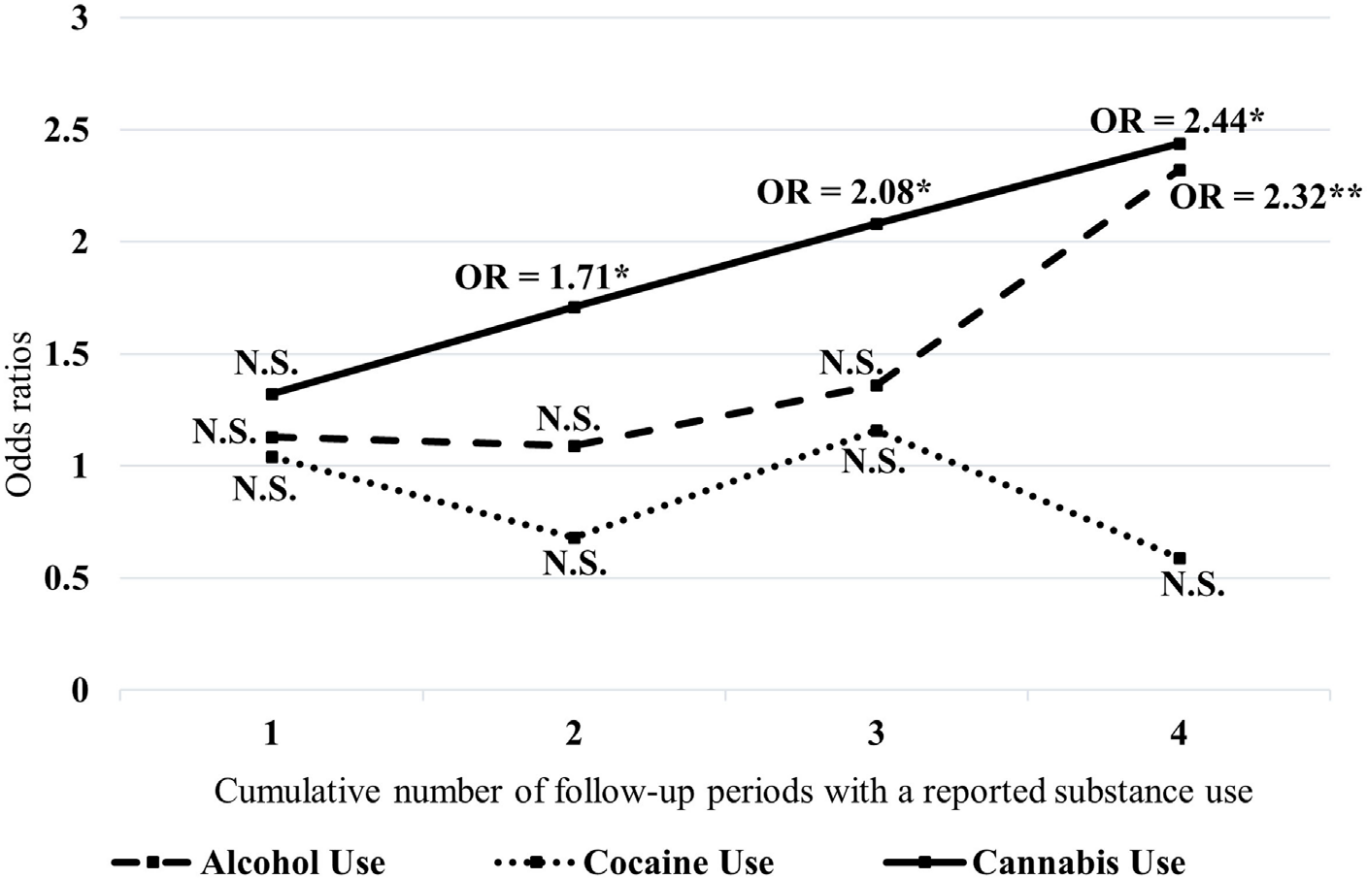
Odds ratios for violent behaviors associated with substance use across each time points. *x*-Axis represent the number of follow-up periods with substance-use, *y*-axis represent the Odds Ratios; Reference is no use of substance across time points; Odds Ratios are controlled for the effects of time, other substances used at each time point, age, age at first hospitalization, sex, ethnicity, Schizophrenia-Spectrum disorders (presence/absence), affective disorders (presence/absence), psychopathic traits (PCL), impulsivity (BIS-11) (*p < 0.05; **p < 0.01; ***p < 0.001; N.S., Not statistically significant).

Moreover, only the continuous use of alcohol over the four follow-up periods significantly increased the odds of future violent behaviors (OR = 2.32, 95% CI: 1.25–4.28, *p* < 0.001). No statistically significant result was observed in the multivariate analysis regarding the cocaine–violence relationship. Figure 1 shows that the odds of having reported a violent behavior augmented significantly as the number of follow-up periods with a reported cannabis consumption increased as well. Moreover, for analyses including potential covariates see Table S2 in Supplementary Material.

Finally, testing for reverse directionality of the relationship between cannabis use and violence showed that violence across time-points was not a statistically significant predictor of cannabis use when controlling for the same covariates included in the preceding model (one time-point: OR = 0.90, 95% CI: 0.64–1.24, *p* = 0.507; two time-points: OR = 1.06, 95% CI: 0.71–1.59, *p* = 0.771; three time-points: OR = 0.81, 95% CI: 0.44–1.49, *p* = 0.500; and four time-points: OR = 0.37, 95% CI: 0.10–1.41, *p* < 0.147).

## Discussion

The primary purpose of this study was to examine the relationship between continued use of cannabis and violence in patients recently discharged from acute psychiatric facilities. Significant findings regarding the adverse effects of cannabis use on violence amid this population were found, notably that the continuity of cannabis usage is a moderate predictor of subsequent violent behavior.

First, we found that the persistency of cannabis use during follow-up periods was associated with an increased risk of subsequent violence. Although we observed that violent behaviors tended to decrease across follow-up periods, the association nevertheless remained statistically significant. As suggested by the authors of the MVRAS (55), this decrement of violence may likely be explained by patients becoming more involved in treatment over time or by an increase of social support. Interestingly, in comparison to the other substances considered in our study (alcohol and cocaine), the persistent use cannabis over time showed a more constant relationship for violence than alcohol and cocaine use. As for the latter substance, while prior literature has shown a cocaine–violence association (3), our results did not confirm such an association possibly due to the lack of cocaine consumers at the different time waves. As depicted in Figure 1, individuals having consumed cannabis at more than 1 time-point were significantly at higher risk of future violent behaviors. In fact, as the number of follow-up periods with a reported substance use increased, the odds of subsequent violence had risen importantly. This was found while controlling for the effect of time, potential time-invariant confounders such as impulsivity and psychopathic traits as well as time-varying covariates, including alcohol and cocaine use. These findings are in line with results from an important longitudinal study amid the general population controlling for the same characteristics as in our study, including antisocial/psychopathic traits, alcohol, and other drug use as well as mental illness (35). Overall, our results imply that patients who reported having used cannabis at each follow-up periods (all four time-points) were 2.44 times more likely to display violent behaviors. This is important as this suggests a moderate relationship between cannabis use and violence.

Second, while there remains ambiguity in the directionality of the association between cannabis use and violence as to whether the use of this drug impacts violence or vice versa, our results pointed toward a unidirectional relationship between its continued use and violence. Hence, contrarily to studies reporting a reciprocal relationship (35, 38–41), we rather found that it was cannabis use that predicted future violent behavior. The reverse relationship was not statistically significant. Therefore, while the association between cannabis and violence found in this study was moderate, persistency of cannabis use across time should be regarded as an indicator of future violent behaviors following psychiatric discharge. This relationship may imply a possible causative role for cannabis use on violence. In fact, past research on the neurobiological effects of chronic cannabis use may aid us to understand the relationship found in our study. For instance, a recent meta-analysis of neuroimaging studies on chronic cannabis users showed structural and functional deficits in the prefrontal cortex mainly associated with inhibitory processing (56). These neural deficits, limiting the ability to suppress a strong tendency to act, are also reported as important components of impulsive aggression (57–59) as well as of antisocial personality disorder and psychopathic traits (60). Although the sequence of these events remains inconclusive, it is plausible that an earlier age of chronic onset of cannabis use (more specifically before brain maturation: prior to 16 years old) could deteriorate neural structures associated with inhibition and, thus, lead to an increased risk of developing adult antisocial behaviors (56, 61–64). This is of particular importance in order to promote awareness about youth substance use and should be targeted by future studies.

### Limitations

While a large longitudinal study design was employed to assess the association between cannabis use and violence amid psychiatric patients, a few limitations are worth to bear in mind. First, the sample size decreased in statistical analyses due to individuals lost across follow-ups and missing data. However, this does not take away the importance of our findings, considering the large sample size remaining. Second, since our hypotheses were not based on the effect of cannabis use on the number of violent acts, we focused solely on the presence of a violent behavior during follow-up periods. Using the MCVI, we employed a single dichotomous variable to assess the presence or absence of a violent behavior across follow-up periods. Thus, we assessed a relatively large definition of the construct. While this surpassed the general scope of our study, it would certainly have been interesting to evaluate the association between cannabis use and specific types of violent behaviors. Some studies have indeed found that cannabis use was associated with certain types of violence (e.g., physical aggression, intimate partner violence) (18, 65). Third, while our initial objective was to observe the effect of cannabis use on the presence of a physical assault at follow-up, our data utilized did not allow us to examine a dose–response relationship, as preliminary evidence has shown amid the general population (32–36). Finally, the data used in our study was self-reported (i.e., violence and substance use). Therefore, future studies should replicate our findings by using alternative methods such as urine toxicology analyses (substance use) as well as hospital/criminal records (violent behaviors). Also, although we included several covariates in our analyses, we did not control for the potential influence of mental state factors as mediators of the cannabis–violence association. Likewise, future studies will need to test the possible effects of psychiatric diagnoses on the relationship between cannabis use and violence. In addition, further research must be conducted on the relationship between cannabis-related characteristics and violence, such as the frequency, the number of cannabis joints smoked, and the cannabis potency ratio [Cannabidiol (CBD)/Delta-9-Tetrahydrocannabinol (THC)], to better understand the association found in this study. Though cannabis potency and synthetic cannabinoid consumption have been considerably increasing since 2000 (66–68), we still nonetheless found that persistency of cannabis use is a relevant risk factor for violence following psychiatric discharge.

## Conclusion

To conclude, our findings are relevant as they aid to shed light on the cannabis–violence association that has been less extensively studied amid psychiatric patients, in whom cannabis use is twice as prevalent in contrast to the general population (44). Compared to prior studies, we employed a prospective design to precisely examine the association between the continuation of cannabis use and violence. Our results are particularly relevant and may have clinical and violence risk management implications as we exposed that the persistency of cannabis use across different time waves was associated with an increased risk of violence in a large sample of patients recently discharged from acute psychiatric facilities. The results from this study show the necessity of further literature on the topic to specify the dose–response relationship with violence. This will have an important impact on preventive strategies to limit the risks of violence associated with cannabis that leads to many major social and health consequences (69).

## Ethics Statement

Please see the MacArthur Violence Risk Assessment Study.

## Author Contributions

AD and SP contributed to the conception of the study. The analysis was ensued by JD and CG. All authors contributed to the interpretation of the data. JD and LD wrote the manuscript. All authors revised the content critically and approved the final version.

## Conflict of Interest Statement

AD discloses HLS therapeutics and SP discloses HLS therapeutics and INSYS therapeutics. The other authors declare no potential conflicts of interest.
