# Supplementary material for: Persistency of Cannabis Use Predicts Violence following Acute Psychiatric Discharge
Source: Front Psychiatry. 2017 Sep 21;8:176. doi: 10.3389/fpsyt.2017.00176 (PMC5613094; doi:10.3389/fpsyt.2017.00176)
Supplement: Supplementary file 1 [file Table_1.PDF]

frontiers

Supplementary Material

Persistency of Cannabis use Predicts Violence following Acute Psychiatric Discharge

Jules R. Dugré; Laura Dellazizzo; Charles-Édouard Giguère; Stéphane Potvin*; Alexandre Dumais*

*** Correspondence:** alexandre.dumais@umontreal.ca; stephane.potvin@umontreal.ca

1 Supplementary Data: Tables

SUPPLEMENTARY TABLE 1

GEE with binomial distribution (log-link) and an unstructured working correlation on subsequent violence with variables entered one at a time (n=592)

	[A]OR (95% CI)	P-value
Cannabis[B]		
Use at 1 time-point	1.60 (1.19-2.15)	0.002
Use at 2 time-points	1.91 (1.27-2.86)	0.002
Use at 3 time-points	2.72 (1.66-4.46)	<0.001
Use at 4 time-points	4.04 (1.99-8.19)	<0.001
Cocaine[B]		
Use at 1 time-point	1.56 (1.12-2.17)	0.009
Use at 2 time-points	1.19 (0.75-1.90)	0.469
Use at 3 time-points	2.27 (1.49-4.80)	0.001
Use at 4 time-points	1.89 (0.75-4.76)	0.177
Alcohol[B]		
Use at 1 time-point	1.42 (1.07-1.89)	0.015
Use at 2 time-points	1.44 (1.02-2.04)	0.039
Use at 3 time-points	1.99 (1.30-3.04)	0.002
Use at 4 time-points	3.39 (1.99-5.78)	<0.001
Psychopathic traits[C]	1.66 (1.37-2.01)	<0.001
BIS-11	1.02 (1.01-1.03)	<0.001
Age	0.97 (0.95-0.99)	0.002
Age at 1st Hospitalization	0.98 (0.96-1.00)	0.047
Men[D]	0.87 (0.66-1.13)	0.293
Caucasian[E]	0.78 (0.58-1.04)	0.087
Schizophrenia-Spectrum Disorders[F]	0.62 (0.45-0.87)	0.006
Affective Disorders[F]	0.96 (0.73-1.25)	0.744

*Note.*BIS-11 : *Baratt Impulsiveness Scale*.
[A] Controlling for the effect of time
[B] Ordinal variable (reference is no use of the substance across time-points)

[C] Ordinal variable (reference group is low psychopathic traits: see (48))
[D] Women as reference
[E] Other ethnicities as reference
[F] Dichotomous variable (Presence/Absence)

SUPPLEMENTARY TABLE 2.

GEE with binomial distribution (log-link) and an unstructured working correlation on subsequent violence adjusted for potential confounders (n=592)

	[A]OR (95% CI)	P-value
Cannabis use[B]		
Use at 1 time-point	1.32 (0.94-1.86)	0.112
Use at 2 time-points	1.71 (1.08-2.70)	0.023
Use at 3 time-points	2.08 (1.16-3.74)	0.025
Use at 4 time-points	2.44 (1.06-5.63)	0.036
Cocaine use[B]		
Use at 1 time-point	1.04 (0.71-1.53)	0.842
Use at 2 time-points	0.68 (0.41-1.14)	0.154
Use at 3 time-points	1.16 (0.59-2.28)	0.674
Use at 4 time-points	0.59 (0.21-1.63)	0.304
Alcohol[B]		
Use at 1 time-point	1.13 (0.84-1.52)	0.420
Use at 2 time-points	1.09 (0.74-1.61)	0.656
Use at 3 time-points	1.36 (0.84-2.21)	0.209
Use at 4 time-points	2.32 (1.25-4.28)	0.007
Psychopathic traits[C]	1.47 (1.19-1.81)	<0.001
BIS-11	1.01 (1.00-1.02)	0.010
Age	0.99 (0.96-1.01)	0.349
Age at 1st Hospitalization	0.99 (0.97-1.01)	0.302
Men[D]	0.73 (0.56-0.96)	0.025
Caucasian[E]	0.81 (0.59-1.10)	0.169
Schizophrenia-Spectrum Disorders[F]	0.59 (0.38-0.92)	0.019
Affective Disorders[F]	0.85 (0.60-1.20)	0.343

Note. BIS-11: Baratt Impulsiveness Scale.
[A] Controlling for the effects of time
[B] Ordinal variable (reference is no use of the substance across time-points)

[C] Ordinal variable (reference group is low psychopathic traits: see (48))
[D] Women as reference
[E] Other ethnicities as reference
[F] Dichotomous variable (Presence/Absence)
